# Physical Activity and Quality of Life Among Caregivers of Children with Duchenne Muscular Dystrophy

**DOI:** 10.3390/healthcare14101425

**Published:** 2026-05-21

**Authors:** Sedat Yiğit, İrem Akgün, Kübra Coşkun, Murat Ali Çınar, Serkan Usgu, Peren Perk

**Affiliations:** 1 Department of Physiotherapy and Rehabilitation, Faculty of Health Sciences, Gaziantep University, 27310 Gaziantep, Türkiye; iremm_akgun@hotmail.com; 2 Institute of Graduate Programs, Department of Physiotherapy and Rehabilitation, Hasan Kalyoncu University, 27500 Gaziantep, Türkiye; kubra.coskun@std.hku.edu.tr; 3 Department of Physiotherapy and Rehabilitation, Faculty of Health Sciences, Hasan Kalyoncu University, 27500 Gaziantep, Türkiye; muratali.cinar@hku.edu.tr (M.A.Ç.); serkan.usgu@hku.edu.tr (S.U.); 4 Department of Pediatric Neurology, Başaksehir Çam and Sakura City Hospital, 34480 Istanbul, Türkiye; perkperen@gmail.com

**Keywords:** Duchenne muscular dystrophy, caregivers, quality of life, physical activity

## Abstract

**Background/Objectives:** Duchenne muscular dystrophy (DMD) is a rare progressive neuromuscular disorder associated with increasing care demands. Despite the critical role of caregivers, their physical activity (PA) levels and health-related quality of life (HRQoL) have not been sufficiently investigated. This study aimed to compare PA levels and HRQoL between caregivers of children with DMD and caregivers of typically developing children. **Methods:** This cross-sectional observational study included 44 individuals: caregivers of children with DMD (n = 22) and caregivers of typically developing children (n = 22). The 36-Item Short-Form Health Survey (SF-36) was used for assessing HRQoL and the International Physical Activity Questionnaire—Short Form (IPAQ-SF) for determining PA levels. **Results:** IPAQ-SF-derived metabolic equivalent of task (MET) values and PA levels were similar between the groups (DMD caregivers: 1744.63 ± 1163.22, controls: 1945.09 ± 1042.12; *p* > 0.05). Caregivers of children with DMD demonstrated significantly poorer scores in several SF-36 domains, including vitality, social functioning, role limitations due to physical problems, bodily pain, and mental health (*p* < 0.05), with the largest difference observed in role limitations due to emotional problems (DMD caregivers: 45.27 ± 28.33, controls: 84.83 ± 24.63; *p* < 0.05). Physical functioning and general health perception scores were comparable (*p* > 0.05). **Conclusions:** Caregivers of children with DMD experience substantial impairments in multiple HRQoL domains, particularly those related to psychosocial well-being and pain, despite comparable PA levels and physical functioning. These findings suggest that reduced HRQoL is not directly explained by PA alone and highlight the need for multidisciplinary interventions targeting psychological health, pain management, and social well-being.

## 1. Introduction

Duchenne Muscular Dystrophy (DMD) is an X-linked recessive neuromuscular disorder caused by mutations in the *Xp21* gene, leading to insufficient or absent dystrophin protein [1]. DMD affects almost exclusively males. It is characterized by progressive muscle fiber degeneration, muscle weakness, delayed ambulation, recurrent falls, Gower’s sign, pseudohypertrophy, loss of gross motor function, and spinal and lower limb deformities, ultimately leading to premature death by the second or third decade of life [2]. Becker muscular dystrophy (BMD), an allelic variant of DMD, has a later onset, milder clinical course, slower progression, and longer life expectancy compared with DMD [3]. The average life expectancy of individuals with BMD is about 40 to 50 years [4]. Dystrophin normally provides mechanical support by stabilizing the cell membrane. Abnormalities in dystrophin are the primary cause of both DMD and BMD. Individuals with BMD retain approximately 10% to 40% of normal dystrophin levels or express a partially functional form of this protein [5]. There is currently no curative treatment for DMD [6] or BMD [7].

The global DMD birth prevalence is 19.8 (95% CI: 16.6–23.6) per 100,000 live male births [8]. Although precise prevalence data for DMD and BMD in Turkey are lacking, it is estimated that approximately 1 in 3500 male children are affected by DMD. In pediatric neurology practice in Turkey, patients with DMD and BMD account for approximately 50% of all children followed for neuromuscular disorders [9]. Early symptoms of DMD typically appear around the age of three, and the diagnosis is suspected by approximately five years of age, when physical abilities divergent from peers become apparent [10]. DMD and BMD may be easier to distinguish based on the age at which patients become wheelchair dependent. Patients with DMD are wheelchair-dependent before the age of 13 years, while individuals with BMD may remain ambulatory beyond the age of 16 years [11]. As the disease progresses, individuals with DMD require increasing assistance in all aspects of daily living, with caregiving responsibilities most often assumed by parents and close family members [12]. This high disease burden contributes to a substantial caregiving population requiring long-term support.

Advances in recent years have enabled individuals with DMD to have a longer life expectancy [13]. However, despite improved survival, DMD remains a disabling, terminal condition that imposes a substantial emotional, physical, and social burden on caregivers, irrespective of disease severity [14,15]. The presence of a chronic illness within the family leads to increased dependence of the affected individual and results in changes to daily life routines [16]. Caregiving involves providing emotional, physical, practical, and economic support to a dependent individual [17]. This process is often associated with psychological distress, reduced sleep quality, impaired social functioning, and diminished quality of life [18]. Caregiving for disabled individuals may prevent caregivers from maintaining a healthy lifestyle and accessing healthcare services [19]. In addition, caregiving for children with DMD has been reported as time-consuming and financially demanding, frequently leading to reduced employment and increased occupational disruption among caregivers [20,21]. Therefore, assessing caregivers’ quality of life is essential to minimize the adverse effects of the caregiving process.

The importance of physical activity (PA) and exercise in the maintenance and promotion of health has been widely recognized by various organizations, particularly the World Health Organization (WHO), who recommend increasing PA levels [22]. The physical and psychological benefits of PA constitute an important factor in supporting caregivers’ health [23]. Caregivers often lack time to engage in health-promoting behaviors such as regular PA, and studies have shown that caregivers participate in less structured PA compared with non-caregivers [24]. Furthermore, given that caregivers are more likely to report adverse health behaviors than non-caregivers such as smoking, poor dietary habits, and insufficient PA [25,26], it is critical to consider health-related outcomes among caregivers of children with physical disabilities.

Existing studies on caregiving burden in chronic pediatric conditions have mainly focused on heterogeneous disability groups rather than disease-specific populations [27,28]. Although the impact of DMD on caregiver burden, mental health, pain, and social functioning has been described [18,29], research specifically addressing caregivers of children with DMD remains scarce in terms of integrated assessment of physical activity levels and health-related quality of life (HRQoL). This represents an important gap, as understanding these two domains together may provide a more comprehensive view of caregiver health status.

In caregivers, physical activity and HRQoL may be interrelated, as reduced physical activity due to caregiving demands may adversely influence HRQoL. Previous studies have demonstrated a positive association between physical activity and various indicators of quality of life; however, these findings have predominantly been derived from research involving older adults or individuals with chronic diseases [30,31].

Caregiver burden in DMD is further compounded by the progressive nature of the disease, leading to increasing dependency over time. As caregiving demands intensify, caregivers may experience musculoskeletal strain, chronic pain, emotional exhaustion, and social withdrawal [29,32,33]. These multidimensional effects highlight the need to evaluate both physical and psychosocial health outcomes in this population.

Based on this gap, the present study aimed to examine physical activity levels and HRQoL in caregivers of children with DMD compared with caregivers of typically developing children. To provide a structured framework for the study, the following research questions were formulated: (1) What are the physical activity levels of caregivers of children with DMD? (2) What is the HRQoL profile of caregivers of children with DMD? (3) Do physical activity levels and HRQoL differ between caregivers of children with DMD and caregivers of typically developing children? We hypothesized that caregivers of children with DMD would demonstrate lower physical activity levels and poorer HRQoL compared with caregivers of typically developing children.

## 2. Materials and Methods

### 2.1. Study Design and Participants

This cross-sectional observational study was conducted between January and February 2026 at the Kübra Coşkun Private Health Professional Services Unit in Osmaniye, located in the eastern Mediterranean region of Turkey. A convenience sampling method was used in this study. The study consisted of two groups: caregivers of children with DMD and caregivers of typically developing children.

Caregivers of children with DMD were recruited through referrals from private special education and rehabilitation centers in Osmaniye and neighboring provinces. Following these referrals, a total of 28 individuals were contacted via telephone or face-to-face interviews and informed about the study. To operationalize the definition of primary caregiver, participants were asked structured questions, including “Who is primarily responsible for the child’s self-care?”, “Who manages the child’s medical follow-up and health appointments?”, and “Who supervises daily activities such as medication administration and nutrition?”. Caregivers of children with DMD were defined as the primary family member who assumed the main responsibility for providing unpaid and ongoing daily care to the child.

Individuals aged 18 years or older who had no communication difficulties and who had been providing care to a family member with DMD aged at least 3 years were eligible for inclusion. Caregivers who were not actively providing care at the time of the study, as well as those with any chronic conditions that could affect HRQoL and/or physical activity levels were excluded. In addition, individuals engaged in professional sports were excluded to avoid potential bias in physical activity measurements. Of the 25 mothers initially identified as primary caregivers of children with DMD, three were excluded due to chronic health conditions (fibromyalgia, diabetes, asthma), resulting in a final sample of 22 caregivers.

Caregivers of typically developing children were recruited among individuals who applied to the same center for consultation and had no children with disabilities or chronic health conditions. Eligibility criteria included being aged 18 years or older, having no communication difficulties, and providing care to a typically developing child. Individuals with chronic conditions that could affect HRQoL and/or physical activity levels and those engaged in professional sports were excluded. No participants were excluded from this group.

All interactions with participants were conducted with respect for privacy and using an empathetic communication approach, and no refusals to participate were encountered.

### 2.2. Ethical Considerations

Approval for the study was obtained from the Institutional Review Board of Hasan Kalyoncu University (No:2025/156; Date: 30 December 2025). Prior to participation, all individuals were provided with detailed information about the nature and scope of the study, and written informed consent was obtained from all participants. Participant data were anonymized prior to analysis. All records were securely stored in a password-protected system with access restricted solely to the research team to ensure confidentiality and data protection.

### 2.3. Study Procedures and Measures

Following the collection of demographic information from caregivers, including age, sex, height, body weight, educational level, and employment status, HRQoL was assessed using the 36-Item Short-Form Health Survey (SF-36) [34]. Physical activity levels of the participants were evaluated using the International Physical Activity Questionnaire—Short Form (IPAQ-SF) [35]. Comparisons were then made between the two groups. Both questionnaires were administered in a standardized environment. Participants did not receive any guidance or assistance during questionnaire completion.

### 2.4. 36-Item Short-Form Health Survey (SF-36)

The SF-36 was originally developed by Ware and Sherbourne [36] as a generic tool for assessing HRQoL, designed to capture both physical and mental health domains. The questionnaire consists of 36 items grouped into eight subdomains: physical functioning (10 items), bodily pain (2 items), role limitations due to physical problems (4 items), mental health (5 items), role limitations due to emotional problems (3 items), social functioning (2 items), vitality (energy and fatigue) (4 items), and general health perceptions (5 items). Items are rated using a Likert-type response scale. Each subdomain is scored on a scale ranging from 0 to 100, with higher scores indicating better health status and lower scores indicating poorer health.

The instrument is widely used in both clinical and research settings due to its brevity, ease of administration, and established measurement properties [34]. It has also been applied in studies involving caregiver populations and chronic disease contexts [37,38]. Reliability and validity of the Turkish adaptation have been demonstrated by Koçyiğit et al. [39]. The SF-36 was selected in the present study due to its multidimensional structure and its ability to evaluate both physical and psychosocial health domains. In comparison with broader quality-of-life instruments such as the WHOQOL-BREF [40], which assess physical, psychological, social, and environmental domains as well as overall quality of life, the SF-36 provides a more health-oriented assessment focusing on perceived health status and functional limitations, making it particularly suitable for evaluating health-related impacts of caregiving. Given that caregiving for children with DMD involves substantial physical demands as well as emotional and social challenges [18,21], the SF-36 was considered appropriate for evaluating HRQoL in this population.

### 2.5. International Physical Activity Questionnaire—Short Form (IPAQ-SF)

The IPAQ-SF was used to assess caregivers’ physical activity levels. It is a self-report questionnaire widely used in clinical and population-based research due to its feasibility and ease of administration [41]. The Turkish version of the IPAQ-SF was used in this study, with its adaptation and application in Turkish populations previously described by Savcı et al. in 2006 [42], and its validity and reliability later confirmed by Sağlam et al. [35].

The IPAQ-SF consists of seven items that assess the frequency and duration of four specific activity types (moderate intensity, vigorous intensity, time spent walking and time spent sitting) performed over the previous seven days. It provides separate scores for vigorous- and moderate-intensity activities and walking, while sitting time is not included in the overall PA score. Total physical activity was calculated as metabolic equivalent of task minutes per week (MET-min/week) according to the IPAQ scoring guidelines [43]. Accordingly, participants were classified into three PA categories: inactive, minimally active, and very active. Participants were categorized as minimally active if they met at least one of the following criteria: engaging in vigorous-intensity physical activity for at least 20 min on at least 3 days per week; performing moderate-intensity activity or walking for at least 30 min on at least 5 days per week; or accumulating at least 600 MET-min/week through a combination of walking and moderate-intensity activities. The very active category included participants who performed vigorous-intensity activity on at least 3 days achieving at least 1500 MET-min/week, or accumulated at least 3000 MET-min/week through a combination of walking, moderate-, or vigorous-intensity activities over seven days. Individuals who did not meet these criteria were classified as inactive [42].

The IPAQ is available in both long (IPAQ-LF) and short (IPAQ-SF) forms for individuals aged 15 to 69 years. While the long form provides a detailed assessment of domain-specific activities, the short form evaluates overall physical activity in a more time-efficient manner [44]. Therefore, to reduce respondent burden and improve feasibility, the IPAQ-SF was preferred in this study.

### 2.6. Statistical Analysis

Statistical analyses were performed using SPSS version 25.0 (IBM Corp., Armonk, NY, USA). The normality of data distribution was checked using the Shapiro–Wilk test. Descriptive data are presented as mean and standard deviation ($\bar{x}$ ± SD) for continuous variables and as number (n) and percentage (%) for categorical variables. Normality was assessed for each continuous variable individually. Based on distribution characteristics, appropriate parametric or nonparametric tests were applied for group comparisons. Between-group comparisons were conducted using the Independent samples *t*-test for variables with a normal distribution and the Mann–Whitney U test for variables with a non-normal distribution. Associations between categorical variables were analyzed using the Chi-square test. Cohen’s d was calculated to quantify the magnitude of the differences between the groups and interpreted using conventional thresholds for effect size, with values of 0.20, 0.50, and 0.80 indicating small, medium, and large effects, respectively [45]. No adjustment for multiple comparisons was applied. An a priori power analysis was conducted using G*Power version 3.1.9.7 [46]. Based on a previously published study with a similar design reporting SF-36 outcomes (Cohen’s d = 0.77) [27], the minimum required total sample size was calculated as 44 participants (n = 22 per group), assuming a statistical power of 80% (1 − β = 0.80) and a significance level of α = 0.05. Although both SF-36 and IPAQ-SF were defined as primary outcomes, the sample size calculation was based on SF-36 outcomes. No separate power analysis was conducted for IPAQ-SF. IPAQ-SF was included to assess physical activity behavior as a complementary outcome alongside HRQoL.

To address potential confounding due to baseline differences in educational level and employment status between groups, a sensitivity analysis using ANCOVA was performed in addition to the primary non-parametric comparisons. In these models, group was included as a fixed factor, while educational level and employment status were entered as covariates to control for their potential confounding effects. Educational level and employment status were dichotomized for analysis. Educational level was categorized as ≤ high school (0) and university or higher (1). Employment status was categorized as not working (0) and working (1), with public and private sector employment combined. Effect sizes were reported as partial eta-squared (η^2^p). SF-36 subdomains included in the ANCOVA were selected based on their clinical relevance to caregiver burden, focusing on mental health, vitality, role limitations due to emotional problems, and bodily pain.

## 3. Results

A total of 44 caregivers participated in the study, including 22 caregivers of children with DMD (age range, 27–48 years) and 22 caregivers of typically developing children (age range, 28–48 years). Among caregivers of children with DMD, 21 were mothers and one was a father, whereas among caregivers of typically developing children, 18 were mothers and four were fathers. All caregivers included in the study were the biological parents of the children. Regarding marital status, the majority of caregivers in both groups were married. Among caregivers of children with DMD, 20 were married and 2 were divorced, whereas among caregivers of typically developing children, 21 were married and 1 was divorced. Caregivers of children with DMD reported providing approximately 6–8 h of care per day, whereas caregivers of typically developing children reported approximately 2–4 h per day. Among caregivers of children with DMD, 20 were responsible for the care of one child, whereas two provided cares for two children. Caregivers of children with DMD reported a total of 24 children with DMD. The mean age of children with DMD was 8.45 ± 3.52 years (range, 4–13), while the mean age of typically developing children was 8.36 ± 3.40 years (range, 3–14).

Regarding household composition, the households of caregivers of children with DMD accounted for a total of 51 children, compared with 43 children in the households of caregivers of typically developing children. The mean total number of children per household was 2.31 ± 0.99 (range, 1–4) in the caregiver group of children with DMD and 1.95 ± 0.72 (range, 1–3) in the caregiver group of typically developing children. In households of children with DMD, the mean number of children with DMD was 1.09 ± 0.29 (range, 1–2), while the mean number of typically developing siblings was 1.22 ± 0.86 (range, 1–3). Of the children with DMD, 16 were ambulatory and 8 were non-ambulatory. Among the ambulatory children, 6 used assistive devices, including ankle-foot orthoses and night splints. Wheelchair use was reported in 5 children, all of whom were classified as non-ambulatory.

The physical characteristics of caregivers of children with DMD and caregivers of typically developing children were comparable (Table 1).

Regarding educational level, 45% of caregivers of children with DMD had a university degree or higher, compared with 86% of caregivers of typically developing children. In terms of employment status, the proportion of unemployed caregivers was higher among caregivers of children with DMD. Significant differences were observed between groups with respect to educational level and employment status (*p* < 0.05) (Table 2).

No significant differences were observed between the groups in total MET-min/week values or IPAQ-SF physical activity levels (*p* > 0.05). Effect sizes for all MET-based comparisons were in the small range (Table 3).

Analysis of HRQoL parameters revealed significant between-group differences in role limitations due to physical problems, role limitations due to emotional problems, vitality (energy/fatigue), mental health, social functioning, and bodily pain, with caregivers of children with DMD reporting lower scores in these domains (*p* < 0.05). Effect sizes were small for the “General health perceptions” domain and ranged from moderate (d = 0.63) to very large (d = 1.49) across the remaining domains (Table 4).

The ANCOVA results examining the effects of educational level and employment status on IPAQ-SF and selected SF-36 subdomains are presented in Table 5. Educational level was significantly associated with the SF-36 bodily pain subdomain (*p* = 0.034, η^2^p = 0.105), indicating a moderate effect size. In contrast, employment status was not significantly associated with bodily pain (*p* = 0.252).

Neither educational level nor employment status showed a statistically significant effect on IPAQ-SF scores (*p* > 0.05). Similarly, no significant effects were observed for the SF-36 subdomains of mental health, role limitations due to emotional problems, or vitality (all *p* > 0.05). Employment status showed non-significant *p*-values approaching the threshold for significance for mental health (*p* = 0.073), role limitations due to emotional problems (*p* = 0.088), and vitality (*p* = 0.076).

## 4. Discussion

This study demonstrated that caregivers of children with DMD experienced significantly greater impairment in several HRQoL domains, including role limitations due to physical and emotional problems, vitality, mental health, social functioning, and bodily pain, compared to caregivers of typically developing children. In contrast, no significant differences were observed in general health perceptions and physical functioning. Additionally, both groups exhibited comparable levels of physical activity. These findings indicate that while overall perceived health status may be preserved, specific functional and psychosocial domains are substantially affected in caregivers of children with DMD.

Caregiver groups were broadly similar in demographic characteristics; however, caregivers of children with DMD were more frequently unemployed, whereas higher educational attainment was more common among caregivers of typically developing children. This pattern is consistent with previous studies reporting lower educational levels and reduced employment rates among caregivers of children with disabilities [47]. Such sociodemographic differences are commonly attributed to the long-term caregiving demands associated with chronic pediatric conditions.

The relationship between sociodemographic factors and health-related outcomes in caregivers remains incompletely understood [48]. Previous studies have suggested that physical activity levels may vary according to socioeconomic and educational status, with individuals of higher educational attainment often reporting greater engagement in physical activity compared with those with lower educational levels [49,50]. In contrast, caregiving responsibilities and time constraints associated with lower socioeconomic conditions may limit opportunities for regular physical activity [51]. However, no significant effects of educational level or employment status on IPAQ-SF scores were observed in the present study. This suggests that within this sample, physical activity levels were not substantially influenced by sociodemographic differences.

Although caregivers of children with DMD differed in educational level and employment status compared with caregivers of typically developing children, these variables did not show a consistent association with physical activity levels or most HRQoL domains in the present analyses. Therefore, the observed differences in HRQoL between groups may not be fully explained by sociodemographic disparities alone. However, the observed association between educational level and bodily pain suggests that socioeconomic factors may still exert domain-specific influences on caregiver health outcomes.

Similarly, no significant associations were found between sociodemographic variables and most HRQoL domains, although educational level showed a significant association with bodily pain, with a moderate effect size. This finding may indicate that higher educational attainment is linked to lower perceived pain, potentially reflecting differences in health literacy, coping strategies, or access to health-promoting behaviors. Employment status was not significantly associated with any HRQoL domains, although non-significant trends were observed for emotional and vitality-related outcomes.

Mothers are generally considered to bear the primary responsibility for childcare [52]. Women often identify with caregiving and accept it as an act of love or a natural and moral duty, consequently assuming responsibility for care [53]. In the present study, although no distinction was made between mothers, fathers, or non-biological caregivers at the inclusion stage, approximately 90% of primary caregivers were mothers, while the remaining 10% were fathers. These findings are consistent with the literature reporting that caregiving roles are predominantly assumed by mothers [54,55].

Having a child may alter parents’ priorities and reduce their engagement in physical activity [56]. This effect appears to be more pronounced among caregivers of children with special needs, who are consistently reported to have lower physical activity levels compared with parents of typically developing children [57,58]. Due to the progressive nature of DMD, caregivers often provide full-time care from childhood through adulthood [21], and as the disease advances, caregiving parents tend to become less physically active [27]. In the present study, a lower proportion of caregivers of children with DMD were classified as very active compared with caregivers of typically developing children (45% vs. 64%). However, identical proportions of participants in both groups met at least the minimally active level (82% vs. 82%), with 18% of participants in each group categorized as inactive. The hypothesis that caregivers of children with DMD would demonstrate lower physical activity levels was therefore not supported by the observed results. These findings indicate that while the proportion of highly active individuals differed between groups, overall physical activity levels based on IPAQ classification thresholds were comparable. Similar findings have been reported in previous studies involving caregivers of children with chronic conditions, where comparable physical activity levels were observed between caregiver and control groups despite differences in health-related outcomes [27,59]. In a large population-based study, Secinti et al. reported that caregivers were more likely than non-caregivers to engage in certain health-promoting behaviors, including physical activity, while also exhibiting greater involvement in health-risk behaviors and poorer overall health outcomes [60]. Although the present study did not examine behavioral mechanisms, these findings provide a broader context for interpreting physical activity patterns in caregiver populations.

The prevalence of PA in the general population has been reported to be 21.4%, with rates of 23.7% in females and 18% in males [61]. In Turkey, data from the Ministry of Health’s “Chronic Diseases and Risk Factors Survey” indicate that 87% of females and 77% of males do not engage in sufficient levels of physical activity [62]. The absence of a significant difference in physical activity levels between groups in our study may reflect broader population-level patterns influencing physical activity participation. These findings suggest that physical activity behavior among women may be shaped not only by caregiving responsibilities but also by a range of personal, social, and societal factors.

Individuals with disabilities often require assistance to perform daily activities, and the continuous caregiving process can negatively affect the quality of life of caregivers [63]. In the presence of a severe chronic disease such as DMD, this burden has a profound impact on both patients and caregivers [64]. The physical burden associated with tasks such as lifting and transferring patients may contribute to musculoskeletal strain and pain [32], which is reflected in the significantly poorer bodily pain and role physical scores observed in caregivers of children with DMD.

The progressive nature of DMD and its associated clinical trajectory impose considerable emotional burden on families. Feelings of loss and fear of death may lead to stress, depression, and lack of energy in caregivers [29,65]. In a comprehensive review by Landfeldt et al., parents and primary caregivers of individuals with DMD were reported to experience high levels of anxiety, emotional stress, and reduced social functioning. Psychological distress was shown to increase with disease progression and escalating caregiving demands and was frequently accompanied by fatigue, sleep disturbance, pain, and social isolation [18]. Studies from Poland and Japan have also shown significantly poorer HRQoL in caregivers of individuals with DMD or BMD compared with population norms, particularly in physical health, social functioning, role limitations, and bodily pain domains [66,67]. Overall, the literature indicates that caregiving in DMD is associated with deterioration in physical health, mental health, and sleep quality [21,29], aligning with the significantly poorer SF-36 outcomes observed in the present study.

### 4.1. Clinical Implications

Considering the multidimensional nature of caregiver burden, physical activity may serve as one component of a broader self-management approach to support caregiver health. However, the present findings suggest that physical activity alone is insufficient to address the full extent of psychosocial and physical challenges experienced by caregivers of children with DMD. Therefore, caregiver support programs may benefit from multimodal strategies that integrate physical activity promotion with psychological support, coping skills training, and structured self-management interventions.

### 4.2. Future Research Directions

These findings raise several important questions for future research regarding the mechanisms underlying caregiver outcomes in DMD. In particular, the dissociation between preserved general health perceptions and marked impairments in specific HRQoL domains warrants further investigation into psychological adaptation, coping processes, and resilience. In addition, the observation of comparable physical activity levels despite substantial caregiving burden suggests that activity behavior may reflect a range of factors beyond caregiving demands, which should be explored in future studies. Further research focusing on psychological and behavioral determinants of caregiver outcomes may help clarify these mechanisms and guide more targeted intervention development.

### 4.3. Limitations

This study has several limitations that should be acknowledged. The cross-sectional design of the study limits the ability to draw causal inferences. The caregivers included in this study were recruited from a single city using a convenience sampling approach, which, together with the relatively small sample size, may limit the generalizability of the findings to broader caregiver populations. A more geographically diverse sample could have strengthened the external validity of the results. In addition, the study groups were not matched, which may have introduced potential baseline differences.

Although basic demographic data for both children with DMD and typically developing children were collected and reported, a direct age-matched or stage-stratified comparison could not be performed. Given the progressive nature of DMD, variability in disease stage and functional status may have influenced caregiver burden and health-related outcomes.

The assessment of physical activity relied on self-reported measures, which may be subject to recall bias, reporting error and overestimation. The use of more objective methods, such as accelerometers, could have provided more precise estimates of physical activity levels. Future studies using longitudinal designs and objective measurement methods are recommended to improve the robustness of findings and allow stronger causal inference. Furthermore, because IPAQ-SF was not included in the a priori sample size calculation, analyses of physical activity outcomes may have been underpowered to detect small between-group differences. Therefore, findings related to physical activity should be interpreted with caution.

Data on marital status were collected and reported descriptively for both groups. The distribution was highly comparable between caregivers of children with DMD (married n = 20, divorced n = 2) and caregivers of typically developing children (married n = 21, divorced n = 1). Given this minimal variability between groups, marital status was not included in inferential analyses, and its potential associations with caregiver outcomes could not be examined in depth.

Anthropometric characteristics, such as body weight and body mass index, were not recorded in children with DMD. These factors may influence the physical demands of caregiving, particularly during lifting, transfers, and mobility assistance. Functional status indicators, including ambulatory status and use of assistive devices, were recorded and reported descriptively in children with DMD but were not included in inferential analyses; therefore, the potential impact of disease severity on caregiver outcomes could not be examined statistically. In addition, although DMD is a relatively rare condition compared with other causes of disability such as cerebral palsy or Down syndrome, studies conducted with larger sample sizes may provide more robust and generalizable conclusions.

## 5. Conclusions

Caregiving for children with DMD is associated with reduced health-related quality of life in specific psychosocial and pain-related domains, particularly role limitations due to emotional problems, while overall physical functioning and physical activity levels remain comparable to those of caregivers of typically developing children. These findings suggest that differences in caregiver outcomes are primarily related to emotional, social, and pain-related aspects rather than global functional capacity. This pattern highlights the need for targeted and multidimensional caregiver support. Multidisciplinary interventions integrating psychological support, coping strategies, and pain management may be beneficial in improving caregiver well-being. Additional supportive approaches, such as physiotherapy-based rehabilitation, relaxation techniques, and individualized exercise programs, may further contribute to overall health maintenance. Future research should adopt multicenter, longitudinal designs and use objective methods to assess physical activity in order to better understand determinants and trajectories of caregiver outcomes in DMD. Such evidence may support the development of more effective and targeted intervention strategies.

## Figures and Tables

**Table 1:** Physical characteristics of caregivers.

	Caregivers of Children with DMD	Caregivers of Typically Developing Children	*p*-Value
	$\bar{x}$ ± SD (n = 22)	$\bar{x}$ ± SD (n = 22)	
**Age (years)**	35.81 ± 6.59	39.18 ± 5.37	0.071 *
**Height (cm)**	164.36 ± 6.49	165.77 ± 9.19	0.560 *
**Body weight (kg)**	71.27 ± 10.2	75.68 ± 16.07	0.248 *
**Body Mass Index (kg/m^2^)**	26.44 ± 3.92	27.57 ± 5.53	0.440 *

DMD, Duchenne Muscular Dystrophy; $\bar{x}$ ± SD, Mean ± Standard deviation, * independent samples *t*-test.

**Table 2:** Comparison of educational level and employment status of caregivers.

	Caregivers of Children with DMD (n = 22)		Caregivers of Typically Developing Children (n = 22)		*p*-Value
**Educational Level**					
Primary school	2	9%	-	-	0.033 *
Secondary school	2	9%	1	5%	
High school	8	37%	2	9%	
University or higher	10	45%	19	86%	
**Employment status**					
Unemployed	13	59%	3	14%	0.007 *
Private sector	2	9%	4	18%	
Public sector	7	32%	15	68%	

DMD, Duchenne Muscular Dystrophy. * *p* < 0.05, Chi-square test.

**Table 3:** Comparison of caregivers’ IPAQ-SF total MET-min/week scores and physical activity levels.

Groups	IPAQ-SF Physical Activity (Total MET-Min/Week) $\bar{x}$ *±* SD	Walking (Total MET-Min/Week) $\bar{x}$ *±* SD	Moderate-Intensity Physical Activity (Total MET-Min/Week) $\bar{x}$ *±* SD	Vigorous-intensity Physical Activity (Total MET-Min/Week) $\bar{x}$ ± SD	IPAQ-SF Physical Activity Category n (%)			
					Inactive	Minimally Active	Very Active	Total
**Caregivers of children with DMD**	1744.63 ± 1163.22	688.50 ± 757.82	332.72 ± 578.65	501.81 ± 719.65	4 (18)	8 (36)	10 (45)	22 (100)
**Caregivers of typically developing children**	1945.09 ± 1042.12	941.23 ± 736.78	323.27 ± 442.26	358.72 ± 482.03	4 (18)	4 (18)	14 (64)	22 (100)
***p*-value**	0.489 ^a^	0.151 ^a^	0.240 ^a^	0.990 ^a^	0.368 ^b^			
***d***	0.18	0.33	0.01	0.23				

IPAQ-SF, International Physical Activity Questionnaire—Short Form; MET, metabolic equivalent of task; DMD, Duchenne Muscular Dystrophy; $\bar{x}$ ± SD, Mean ± Standard deviation; d, Cohen’s d. ^a^ Mann–Whitney U Test, ^b^ Chi-square test.

**Table 4:** Comparison of SF-36 scores between caregiver groups.

SF-36 Subdomains	Caregivers of Children with DMD (n = 22)	Caregivers of Typically Developing Children (n = 22)	*p*-Value	*d*
	$\bar{x}$ ± SD	$\bar{x}$ ± SD		
**Physical functioning**	68.18 ± 29.66	83.18 ± 15.77	0.096 ^a^	0.63
**Role limitations due to physical problems**	56.93 ± 32.60	80.22 ± 30.37	0.019 ^a,^*	0.73
**Role limitations due to emotional problems**	45.27 ± 28.33	84.83 ± 24.63	<0.001 ^a,^*	1.49
**Vitality (energy and fatigue)**	42.27 ± 21.14	69.77 ± 24.02	<0.001 ^b,^*	1.21
**Mental health**	54.90 ± 21.20	68.18 ± 18.24	0.032 ^b,^*	0.67
**Social functioning**	55.68 ± 22.06	80.11 ± 19.91	<0.001 ^b,^*	1.16
**Bodily pain**	53.97 ± 26.09	70.90 ± 24.36	0.037 ^a,^*	0.69
**General health perceptions**	59.09 ± 17.22	64.31 ± 20.13	0.360 ^b^	0.27

SF-36,36-Item Short-Form Health Survey; DMD, Duchenne Muscular Dystrophy; $\bar{x}$ ± SD, Mean ± Standard deviation; d, Cohen’s d. * *p* < 0.05, ^a^ Mann–Whitney U test, ^b^ Independent-Samples *t*-test.

**Table 5:** Effects of educational level and employment status on IPAQ-SF and selected SF-36 parameters (ANCOVA).

**IPAQ-SF**	**F**	***p***	**η^2^p**
Educational Level	2.265	0.140	0.052
Employment Status	2.209	0.145	0.051
**SF-36-Mental Health**	**F**	***p***	**η^2^p**
Educational Level	0.002	0.967	0.000
Employment Status	3.388	0.073	0.078
**SF-36-Role limitations due to emotional problems**	**F**	***p***	**η^2^p**
Educational Level	0.564	0.457	0.014
Employment Status	3.057	0.088	0.069
**SF-36-Bodily Pain**	**F**	***p***	**η^2^p**
Educational Level	4.835	0.034	0.105
Employment Status	1.348	0.252	0.032
**Vitality**	**F**	***p***	**η^2^p**
Educational Level	3.382	0.073	0.076
Employment Status	3.312	0.076	0.075

IPAQ-SF: International Physical Activity Questionnaire—Short Form; η^2^p: partial eta-squared.

## Data Availability

The data that support the findings of this study are available from the corresponding author (SY) upon reasonable request. The data are not publicly available due to ethical restrictions and the need to protect the confidentiality of the study participants.
